# Detection of Rice Prolamin and Glutelin Content Using Hyperspectral Imaging Combined with Feature Selection Algorithms and Multivariate Regression Models

**DOI:** 10.3390/foods14193304

**Published:** 2025-09-24

**Authors:** Chu Zhang, Zhongjie Tang, Xiaojing Tan, Hengnian Qi, Xincheng Zhang, Shanlin Ma

**Affiliations:** 1 School of Information Engineering, Huzhou University, Huzhou 313000, China; chuzh@zjhu.edu.cn (C.Z.);; 2 School of Life and Health Sciences, Huzhou College, Huzhou 313000, China; 3 Institute of Crop Science, Huzhou Academy of Agricultural Sciences, Huzhou 313000, China

**Keywords:** hyperspectral imaging, prolamin, glutelin, deep learning, feature selection

## Abstract

Prolamin and glutelin are the major constituents of rice protein. The rapid and non-destructive detection of prolamin and glutelin content is conducive to the accurate assessment of rice quality. In this study, hyperspectral imaging combined with regression models and feature wavelength selection was employed to detect the rice prolamin and glutelin content. Feature wavelength selection was achieved using the successive projections algorithm (SPA), competitive adaptive reweighted sampling (CARS), and convolutional neural network (CNN)-based Gradient-weighted Class Activation Mapping++ (GradCAM++). Partial least squares regression (PLSR), support vector regression (SVR), back-propagation neural network (BPNN), and CNN models were established using the full spectra and the feature wavelengths. The BPNN models showed the best prediction performance for prolamin and glutelin. The optimal BPNN models achieved a correlation coefficient (*r*) greater than 0.8 for both proteins. Performance differences were observed between models using feature wavelengths and those using the full spectra. The GradCAM++ method was used to select feature wavelengths with different threshold values, and the performance of different threshold values were compared. The results demonstrated that hyperspectral imaging with multivariate data analysis was feasible for predicting the rice prolamin and glutelin content. This study provided a methodological reference for detecting prolamin and glutelin in rice, as well as the other protein types.

## 1. Introduction

Rice is not only a primary source of calories but also a significant nutritional source for over half of the global population [1]. High-protein rice may enhance human nutrition for impoverished households, particularly in regions where rice is a staple food. Therefore, increasing the content of soluble proteins has become an important breeding objective to improve the nutritional quality of rice [2]. Consequently, enhancing the nutritional value of rice is crucial for improving the health of rice consumers. Prolamin and glutelin are the two main types of proteins in rice grains. The content of prolamin and glutelin in rice vary significantly, with glutelin accounting for 60–80% of the total protein content, and prolamin accounting for 5–25% [1].

The traditional methods for measuring prolamin and glutelin content include High-Performance Liquid Chromatography (HPLC) [3] and Coomassie Blue G250 [1]. However, these methods require expensive equipment and skilled technicians. Additionally, traditional analytical methods often necessitate preprocessing steps such as drying, polishing, milling, and grinding [4]. Therefore, there is a need for a rapid and non-destructive form of technology to measure the protein content of rice during the harvesting and storage stages. Hyperspectral imaging (HSI) technology, as a rapid, non-destructive, and efficient detection method, has demonstrated significant potential in the field of agricultural product quality inspection. This method provides the concurrent spatial and spectral imaging of objects, supporting the comprehensive evaluation of external and internal quality. By analyzing the unique spectral features of materials, the effectiveness of HSI in agricultural and food quality detection has been widely validated [5,6]. Moreover, extensive research has been conducted on the application of hyperspectral imaging for crop seed quality inspection [7]. In the context of rice quality detection, researchers have employed hyperspectral imaging technology to study the starch and protein content in rice [8,9,10,11]. Generally, the prediction of the crude protein content or total protein content in rice is studied [9,10], and the prediction of the content of certain types of protein in rice is rarely studied. A previous study had used near-infrared spectroscopy to the detect prolamin and glutelin content in rice [12]. Currently, there are no published studies that apply hyperspectral imaging technology to measure the prolamin and glutelin content in rice. Thus, it is worth investigating the determination of the prolamin and glutelin content in rice using hyperspectral imaging.

Spectral-data-based modeling is the cornerstone of HSI technology for food quality detection. Traditional machine-learning algorithms, such as Partial Least Squares Regression (PLSR), Support Vector Regression (SVR), and Artificial Neural Networks (ANNs), have been widely applied in spectral-data-based modeling and analysis [13], including seed quality detection [14]. Deep learning (DL) utilizes multi-layer neural networks to automatically learn hierarchical features from data, characterized by end-to-end learning, powerful representational capabilities, and a reliance on large-scale data and computational resources. Notable deep-learning architectures include Convolutional Neural Networks (CNNs), Recurrent Neural Networks (RNNs), and Transformers. Deep learning has been widely applied in the field of spectral data analysis and has proven to be a highly effective method for spectral data analysis [15]. Extensive research has also been conducted on deep-learning algorithms for seed quality detection, achieving promising results [16].

HSI generates substantial amounts of data. Therefore, selecting informative spectral bands from HSI data for dimensionality reduction is crucial in hyperspectral data analysis [17]. Feature wavelength selection methods effectively enhance the modeling efficiency and prediction accuracy [18]. Researchers have proposed numerous feature wavelength selection algorithms to reduce the data dimensionality while improving the performance of predictive models [19,20,21,22]. In deep-learning-based spectral data analysis, interpreting the importance of spectral bands is essential. Several methods have been applied to assess spectral feature importance in deep-learning models, such as Class Activation Mapping (CAM), Gradient-weighted Class Activation Mapping++ (GradCAM++), SHapley Additive exPlanations (SHAP), and local interpretable model-agnostic explanations (LIME) [23,24,25,26,27]. Wavelengths with a high contribution to the task can be regarded as feature wavelengths and subsequently used for feature-based modeling [26].

This study aimed to explore the feasibility of using hyperspectral imaging (HSI) technology to predict the prolamin and glutelin content in rice. This study innovatively combined hyperspectral imaging with feature selection algorithms and machine- and deep-learning methods to detect the content of prolamin and glutelin in rice. The specific research objectives were to (1) develop predictive models (PLSR, SVR, Backpropagation Neural Networks (BPNNs), and CNNs) to estimate the prolamin and glutelin content, respectively; (2) apply feature selection algorithms (SPA, CARS, and Grad-CAM++) to identify key spectral features; and (3) construct optimized models (PLSR, SVR, BPNNs, and CNN) for the prolamin and glutelin content prediction using the full spectra and the selected features.

## 2. Materials and Methods

### 2.1. Sample Sources

The experiments and sample preparation had been described in detail in our previous study for rice milling quality detection [28]. Two japonica rice varieties (Xiushui 121 and Zhehujing 26) were used for field experiments conducted in 2023 at the Balidian Experimental Station of Huzhou Academy of Agricultural Sciences and Jianliang Family Farm in Huzhou, China. The experimental design adopted nitrogen fertilization as the main plot factor and variety as the subplot factor, with 16 treatment combinations established. Additional fertilization was applied during the tillering, panicle initiation, and booting stages. At maturity, samples were systematically collected and subjected to standardized processing for hyperspectral image acquisition and prolamin and glutelin content determination, providing a reliable data foundation for subsequent quality analysis. A total of 302 samples were obtained for analysis, and each sample contained 20 g of rice seeds.

### 2.2. Hyperspectral Image Acquisition and Spectral Extraction

The hyperspectral image acquisition and spectral data extraction procedures had been described in detail in our previous study [28]. The hyperspectral imaging setup included several key components: a hyperspectral camera, an illumination source, a movable stage, and a control computer. Specifically, the system employed a hyperspectral camera (FX17, SPECIM, Oulu, Finland) capable of capturing spectral data across the near-infrared wavelength range of 900–1700 nm.

To eliminate noise interference caused by instrumental and environmental factors during measurement, the raw spectral data were preprocessed. Prior to spectral extraction, the noise bands at both ends of the spectra were selectively removed based on a preview of the seed spectra. As in our previous study [28], the first 13 spectral bands (below 980 nm) at the beginning of the spectra and the last 11 bands (above 1684 nm) at the end of the raw spectra were excluded. This resulted in an effective spectral range of 980–1684 nm for further analysis, comprising 200 spectral bands.

### 2.3. Determination of Prolamin and Glutelin Contents

**Figure 1:**
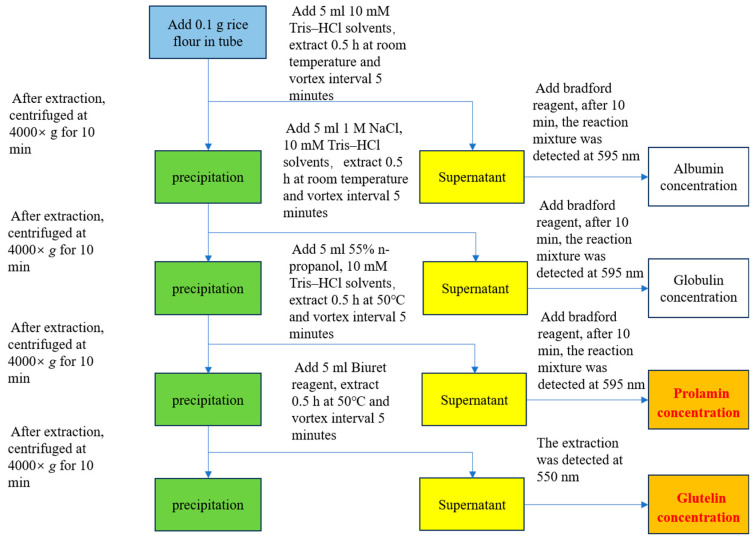
Summary diagram of the sequential extraction process for prolamin and glutelin in rice and the corresponding measurement procedure.

Rice albumin, globulin, prolamin, and glutelin can be extracted and measured by the methods of Ning et al. [29] with minor modifications. Briefly, four protein fractions were sequentially extracted from 0.1 g rice flour in the 5 mL solvents of 10 mM Tris–HCl (Solarbio, Beijing, China), pH 7.5 (albumin); 1 M NaCl (Sinopharm, Shanghai, China), 10 mM Tris–HCl, pH 7.5 (globulin); 55% n-propanol (Sinopharm, Shanghai, China), 10 mM Tris–HCl, pH 7.5 (prolamin); and 0.24% CuSO_4_ (Sinopharm, Shanghai, China), 1.68% KOH (Sinopharm, Shanghai, China), 0.5% potassium sodium tartrate (Sinopharm, Shanghai, China), and 50% iso-propanol (Sinopharm, Shanghai, China) (glutelin), respectively. The extract was centrifuged at 4000× *g* for 10 min using a centrifuge (TD-420, Shuke, Chengdu, China). The former three supernatants were determined using the Bradford reagent method for corresponding protein content (at 595 nm), and the last one was determined by the biuret method for glutelin content (at 550 nm) by a microplate reader (Epoch, BioTek, Winooski, VT, USA). Here, we paid attention to prolamin and glutelin content because they are the major storage proteins affecting eating quality and nutritional quality. The extraction process flowchart is shown in Figure 1.

### 2.4. Regression Analysis Methods

#### 2.4.1. Machine-Learning Methods

Partial Least Squares Regression (PLSR) [30] is a statistical and machine-learning technique used for multivariate data analysis. It builds predictive models by finding the best linear relationship between independent and dependent variables, and is particularly suitable for spectral data analysis. PLSR focuses on maximizing the covariance between predictors and response variables to reduce prediction errors. The algorithm iteratively performs regression and data compression, generating a set of orthogonal latent factors that serve as principal components. These components are then validated using a calibration dataset. In this research, the optimal model parameters were determined through a grid search approach combined with ten-fold cross-validation, with the number of principal components being fine-tuned between 1 and 20.

Support Vector Regression (SVR) [31], as an extended form of Support Vector Machine in regression problems, has a wide range of applications in the field of spectral data analysis. This method achieves data regression by constructing the optimal hyperplane, and its core advantage lies in its excellent performance in processing high-dimensional nonlinear spectral data. SVR can flexibly adapt to different data distribution characteristics with the help of kernel functions. In the process of parameter optimization, the study adopted a strategy of grid search combined with ten-fold cross-validation. Various kernel functions, such as linear kernel, radial basis function (RBF), and polynomial kernel (Poly), were used. The regularization parameter range was set to the order of 10^−7^ to 10^7^. The kernel function coefficients were set to two modes, “scale” and “auto”, for comparative optimization.

Backpropagation Neural Network (BPNN) [32], a well-known multilayer feed-forward neural network architecture, finds broad application in domains ranging from pattern recognition to regression analysis. The BPNN employs the Backpropagation algorithm to update weights and biases, minimizing the Mean Squared Error (MSE) loss to enhance model performance. BPNN takes feature vectors of hyperspectral data as input, extracts spectral features through nonlinear transformations in multiple hidden layers, and predicts prolamin and glutelin content in the output layer. This study used trial-and-error approach to optimize BPNN, and the model parameters of BPNN (such as the number of hidden layers, the number of neurons in the hidden layers, and the learning rate, etc.) were determined through continuous debugging to obtain the optimal results. Due to the randomness of the BPNN model, the models were run at least five times to evaluate the performance of the parameters. By comparing model performance under different parameters, the models with optimal results were saved and used for further analysis. After trial and error, the BPNN model consisted of an input layer, a hidden layer, and an output layer. The input layer size was 200, and the hidden layer size was 1024. The training parameters included the number of epochs of 5000, a batch size of 16, and a learning rate (LR) of 0.001. The hidden layer employed the Rectified Linear Unit (ReLU) activation function, and the Adam optimizer was used to enhance regression prediction accuracy. For BPNN, models were built based on the training set, and the predictive performance of the model was evaluated based on the results of the validation set to explore the optimal model parameters.

#### 2.4.2. Deep-Learning Methods

Convolutional Neural Network (CNN) [33] is a class of deep-learning models specifically designed for processing grid-structured data (e.g., spectral data). By extracting deep-level features of hyperspectral data layer by layer, CNN learns the nonlinear mapping relationship between spectral features and target indicators, ultimately achieving precise regression prediction. Its typical architecture consists of an input layer, convolutional layers, pooling layers, fully connected layers, and an output layer, forming a hierarchical feature learning system. CNN can exhibit stronger nonlinear modeling capabilities, enabling automatic extraction of complex features from high-dimensional spectral data, thereby improving the accuracy and robustness of rice prolamin and glutelin content prediction.

In this study, the CNN model was built based on the PyTorch framework, with ReLU used as the activation function in the convolutional layers. This study used trial-and-error approach to tune CNN, and the model parameters of CNN (such as the model architecture, the learning rate and the epochs, etc.) were determined through continuous debugging to obtain the optimal results. Due to the randomness of the CNN model, the models were run at least five times to evaluate the performance of the parameters. By comparing model performance under different parameters, the models with optimal results were saved and used for further analysis. After trial and error, the CNN model for predicting rice prolamin and glutelin content consisted of five layers, including two convolutional layers and three fully connected layers. The overall architecture of the CNN model was the same as our previous study [28]. The batch size was set to 128, the learning rate (LR) was set to 0.001, and the Adam algorithm was employed to minimize the loss during model training. For CNN, models were built based on the training set, and the predictive performance of the model was evaluated based on the results of the validation set to explore the optimal model parameters.

### 2.5. Feature Selection Algorithms

#### 2.5.1. Successive Projections Algorithm (SPA)

SPA is an efficient feature wavelength selection method, particularly suitable for modeling tasks with one-dimensional spectral data [34]. SPA minimizes collinearity among wavelength variables to identify the most informative and least redundant feature subset from full-spectra data. Especially valuable for small-sample spectral analysis, SPA eliminates redundant variables and reduces model complexity.

#### 2.5.2. Competitive Adaptive Reweighted Sampling (CARS) Algorithm

CARS [35] is a feature wavelength selection method based on Darwinian evolutionary theory, specifically designed for variable selection in multivariate calibration. This algorithm effectively reduces redundancy in high-dimensional data such as hyperspectral datasets. By iteratively eliminating non-informative variables and dynamically weighting retained variables [36], CARS aims to enhance the predictive performance of selected wavelengths.

The implementation of CARS involves the following key steps: First, Monte Carlo sampling is employed to generate multiple data subsets containing random wavelength combinations. Subsequently, a linear regression model is built for each subset, with wavelength importance evaluated based on the absolute values of regression coefficients (higher coefficients indicate greater informational value). Next, a competitive mechanism progressively eliminates low-importance wavelengths. Finally, retained wavelengths undergo adaptive reweighting to reinforce critical features. This iterative process continues until the optimal wavelength combination is determined.

#### 2.5.3. Gradient-Weighted Class Activation Mapping++ (GradCAM++)

Grad-CAM++ is an enhanced convolutional neural network (CNN) visualization interpretation method that improves localization capability for modeling decision-critical regions through higher-order gradient weighting [37]. Building upon the original Grad-CAM approach, it incorporates second- and third-order gradient information to compute spatially sensitive weights, enabling more precise identification of important features in multi-object instances or dispersed patterns. The generated heatmaps reveal core regions in the data that influence classification results more clearly, and no model architecture modifications are required, which makes it applicable for enhancing interpretability in classification, detection, and similar tasks. In this study, we adapted the original Grad-CAM++ (designed for 2D image analysis) into a 1D spectral data analysis version for regression. This modified Grad-CAM++ determined the importance of each individual wavelength, allowing feature wavelength selection based on importance thresholding.

### 2.6. Software and Hardware

All data processing and model development were conducted on a computer system equipped with 16 GB RAM, an NVIDIA GeForce RTX 4060 GPU (Santa Clara, CA, USA), and an Intel i7-12650 CPU.

The computational environment utilized Python (v3.8), with PyCharm Community Edition 2024.1 serving as the programming platform. Data extraction was implemented in Python, while machine-learning algorithms were executed using scikit-learn 1.3.0 and PyTorch 1.9.1. Deep-learning implementations were achieved through the PyTorch framework.

### 2.7. Evaluation Metrics

To systematically assess the predictive performance of the four models used in this study (PLSR, SVR, CNNs, and BPNNs) for rice prolamin and glutelin content, two core evaluation metrics were adopted: the correlation coefficient (*r*) between predicted and actual values, and the root mean square error (RMSE) between predicted and actual values. The *r* measures the model’s goodness of fit, while the RMSE evaluates the model’s prediction accuracy.

For each of the four models (PLSR, SVR, CNNs, and BPNNs), these two metrics were calculated on the training set, validation set, and test set. An *r*-value closer to 1 indicates better model fitting, while an RMSE closer to 0 signifies smaller prediction errors. A model is considered superior when it simultaneously achieves both a higher *r*-value and a lower RMSE.

## 3. Results

### 3.1. Outlier Removal and Dataset Partitioning

The spectral profiles of rice samples used in this study were presented in our previous research [28]. A total of 302 samples were initially included, with both the prolamin and glutelin content measured for each sample. Prior to the modeling analysis, PLSR models were first established based on all sample spectra along with their respective prolamin and glutelin content to identify and remove samples with significant prediction errors, thereby reducing the negative impact of extreme samples on model performance [28]. In this study, a total of 288 samples were retained for both prolamin and glutelin content prediction research. For both the prolamin and glutelin content prediction, the remaining 288 samples were divided into training, validation, and test sets at a ratio of 4:1:1, specifically comprising 192 samples for training, and 48 samples each for validation and testing. Notably, there was no sample overlap among these three datasets. Table 1 presents the statistical information of the prolamin and glutelin content across the three datasets.

### 3.2. Results of Full-Spectra-Based Models for Prolamin and Glutelin Content Prediction

**Figure 2:**
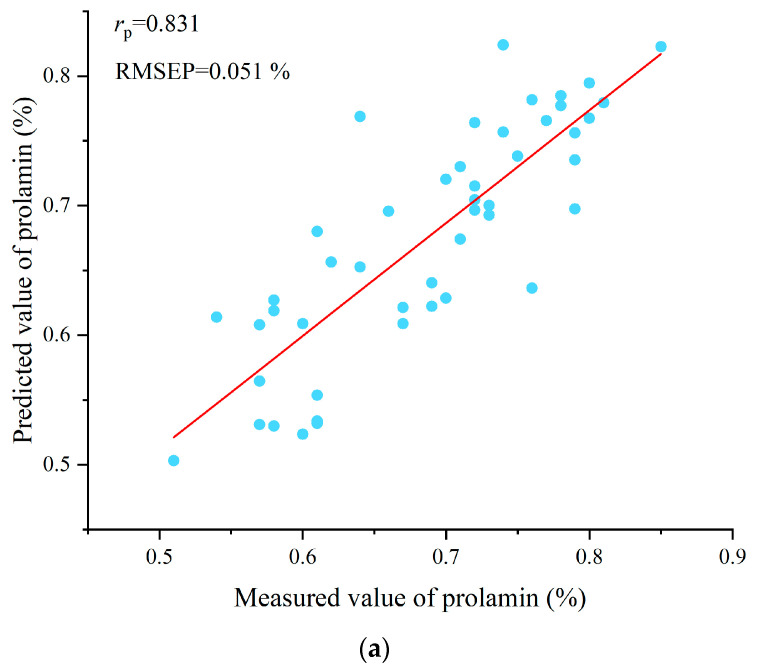

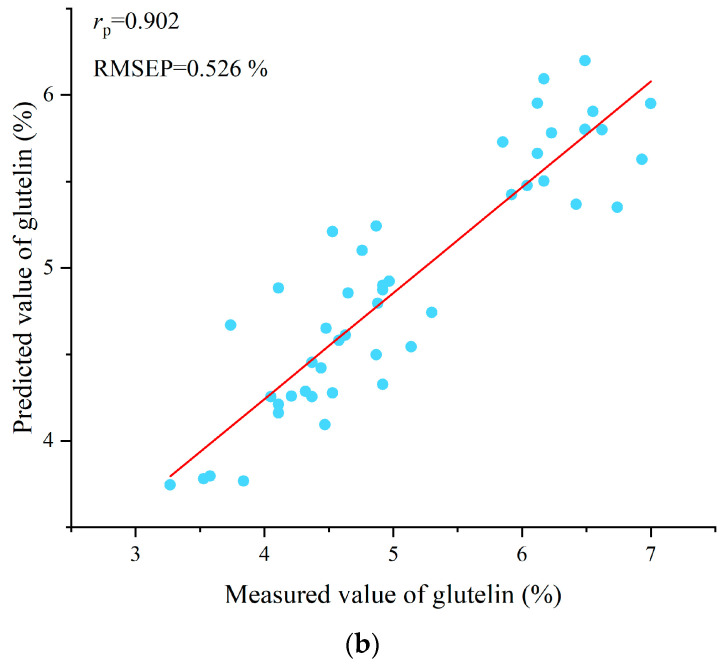
(**a**) Scatter plot of measured and predicted prolamin content in test set of BPNN model; and (**b**) scatter plot of measured and predicted glutelin content in test set of BPNN model. The red lines are the linear fit lines between the measured values and the predicted values. The scatter plots are used to visually present the linear fit performance between the measured values and the predicted values. These scatter plots indicated that BPNN models can be used to predict prolamin and glutelin content in rice using full spectra, and there is room for further improvement.

Based on the preprocessed datasets for prolamin and glutelin, PLSR, SVR, BPNN, and CNN models using the full spectra were developed, with their performance metrics summarized in Table 2. For the prolamin content prediction, the BPNN model achieved the best performance, followed by PLSR and CNN, while the SVR model showed relatively inferior results. All models obtained correlation coefficients above 0.6 across the three datasets. Regarding the glutelin prediction, the BPNN model outperformed its CNN, PLSR, and SVR counterparts, with all models exhibiting correlation coefficients exceeding 0.8, except for the PLSR prediction set (*r* = 0.794). The scatter plots of the test set predicted by BPNN for both prolamin and glutelin are presented in Figure 2, respectively. Overall, the full-spectra models demonstrated a better predictive capability for glutelin content compared to prolamin, confirming the feasibility of combining hyperspectral imaging with machine-learning and deep-learning approaches for the quantitative detection of rice prolamin and glutelin content.

### 3.3. Results of Prolamin and Glutelin Content Detection Models Based on Characteristic Wavelengths

#### 3.3.1. Prediction of Prolamin and Glutelin Content Using SPA-Selected Characteristic Wavelengths

For predicting the prolamin and glutelin content, this study first employed the characteristic wavelengths identified by SPA. The number of characteristic wavelengths selected for both prolamin and glutelin was set within the range of 30–50. Ultimately, 30 characteristic wavelengths were selected for each protein (prolamin and glutelin), with the results presented in Table 3.

**Figure 3:**
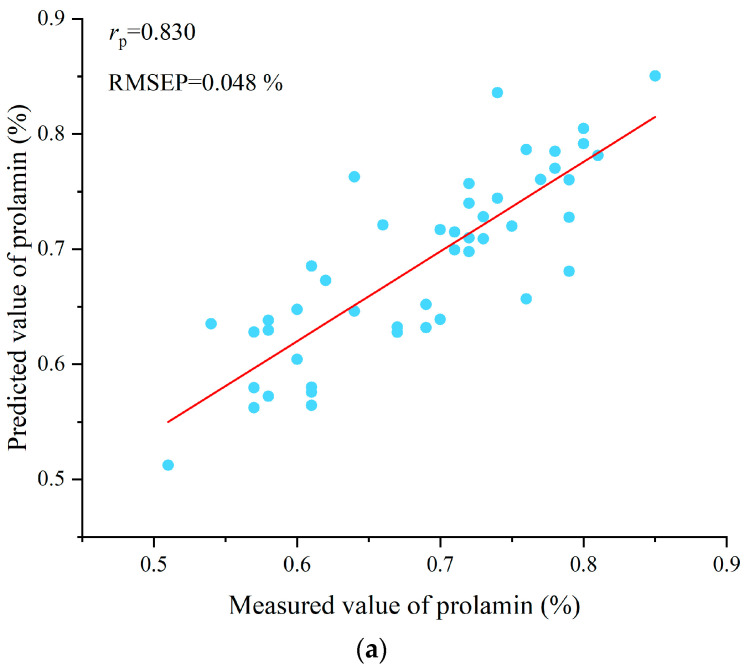

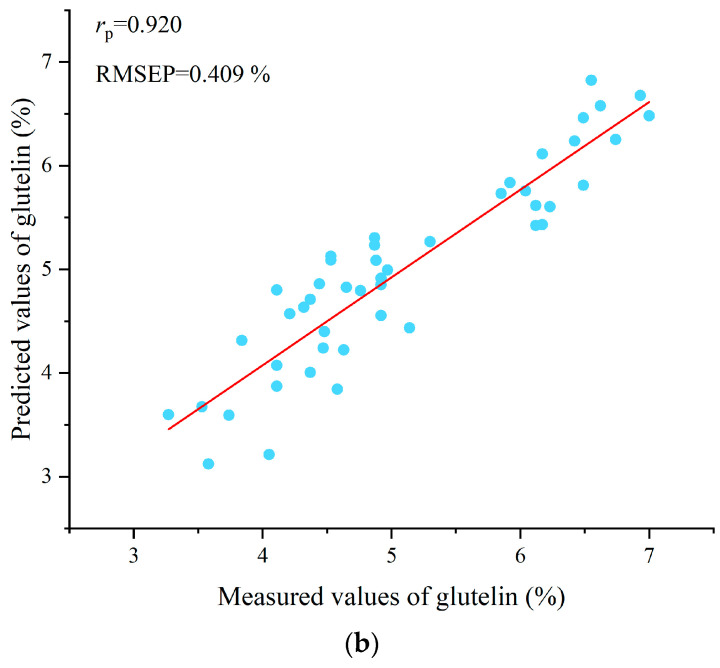
(**a**) Scatter plot of prolamin content in BPNN test set using SPA-selected characteristic wavelengths; and (**b**) scatter plot of glutelin content in BPNN test set using SPA-selected characteristic wavelengths. The red lines are the linear fit lines between the measured values and the predicted values. The scatter plots are used to visually present the linear fit performance between the measured values and the predicted values. These scatter plots indicated that BPNN models can be used to predict prolamin and glutelin content in rice using full spectra, and there is room for further improvement.

The prediction models of PLSR, SVR, the BPNN, and the CNN were established based on the characteristic wavelengths selected by SPA, and their results are presented in Table 4. An analysis of Table 4 reveals that, for prolamin content prediction, the BPNN model achieved the optimal performance with correlation coefficients exceeding 0.8 across all datasets (the training, validation, and test sets). While PLSR and CNNs showed relatively inferior results compared to BPNNs, they still maintained a satisfactory performance with correlation coefficients above 0.7 for all datasets. The SVR model demonstrated the poorest predictive capability among all models. Regarding glutelin content prediction, BPNNs exhibited the best performance followed by CNNs, with both models achieving correlation coefficients greater than 0.8 across all datasets. PLSR and SVR showed a comparable predictive accuracy. Figure 3 displays the scatter plots of the BPNN test set predictions for prolamin and glutelin content using the SPA-selected characteristic wavelengths. These comprehensive results confirmed that the characteristic wavelengths selected by the SPA algorithm were effective for predicting the rice prolamin and glutelin content.

#### 3.3.2. Prediction Study of Prolamin and Glutelin Content Based on CARS-Selected Characteristic Wavelengths

The study additionally employed the CARS algorithm for characteristic wavelength selection, with parameter settings of 5000 Monte Carlo sampling runs, a maximum of 20 principal components, and leave-one-out cross-validation. Table 5 presents the CARS-selected characteristic wavelengths for prolamin and glutelin.

The subsequent development of the PLSR, SVR, BPNN and CNN models using these wavelengths yielded the results shown in Table 6. For prolamin prediction, the BPNN model demonstrated superior performance followed by CNNs, while SVR showed the weakest results with correlation coefficients below 0.7 across all the three datasets, and PLSR exhibited some overfitting. In glutelin prediction, BPNNs again achieved the optimal results, with SVR ranking second, both maintaining correlation coefficients above 0.8, whereas CNNs performed relatively poorly. The overall results demonstrated that the characteristic wavelengths selected by the CARS algorithm could be effectively applied for predicting the rice prolamin and glutelin content.

#### 3.3.3. Analysis of Results Combining Grad-CAM++ with Conventional Methods

**Figure 4:**
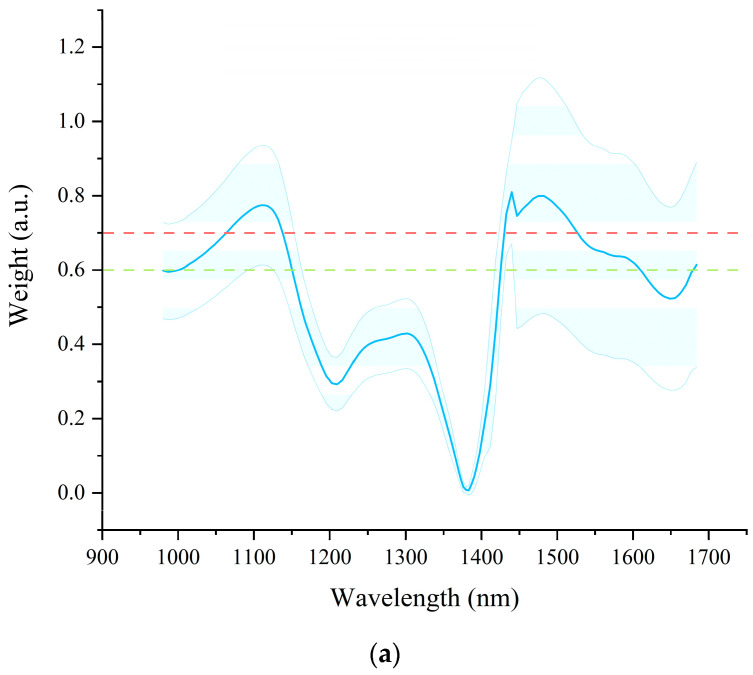

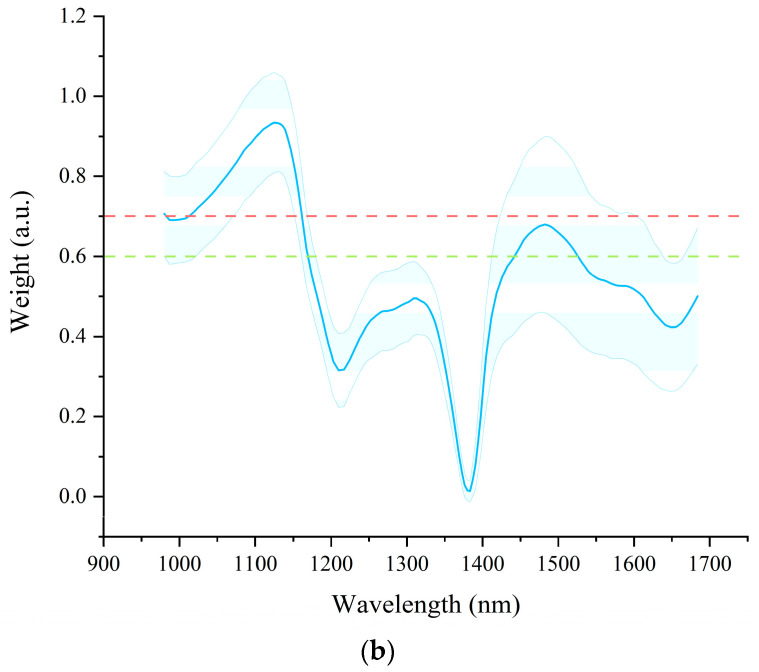
(**a**) Weight distribution obtained by Grad-CAM++ of the full-spectra CNN model for prolamin content prediction; and (**b**) weight distribution obtained by Grad-CAM++ of the full-spectra CNN model for glutelin content prediction. The solid lines represent the average values of the weight of each wavelength of the samples in the test set; the corresponding shading areas indicate the standard deviation of the weight values of each wavelength. The dot lines with different colors indicated different threshold values for feature wavelength selection. The wavelengths with average weight values larger than the threshold value are identified as the feature wavelengths.

In this study, Grad-CAM++ computations were performed using the test set of the full-spectra CNN model, specifically targeting the second convolutional layer to generate heatmaps and activation values for both prolamin and glutelin. The obtained weights were normalized, with the results visualized in Figure 4. Since no established standards currently exist for determining the importance thresholds, this study selected features with average weight value means exceeding 0.6 and 0.7 to investigate how different importance thresholds affect the prediction of the prolamin and glutelin content. The number of characteristic wavelengths selected for prolamin with a weight value greater than 0.6 was 98, covering the wavelength ranges of 1001–1147, 1421–1606, and 1677–1681 nm. The number of characteristic wavelengths selected with a weight value greater than 0.7 was 48, covering the wavelength ranges of 1064–1134 and 1428–1521 nm. The number of characteristic wavelengths selected for glutelin with a weight value greater than 0.6 was 78, covering the wavelength ranges of 981–1165 and 1439–1521. The number of characteristic wavelengths selected with a weight value greater than 0.7 was 48, covering the wavelength range of 981–1158.

Subsequently, the PLSR, SVR, BPNN, and CNN models were developed based on the characteristic wavelengths identified by Grad-CAM++. The regression analysis results using a wavelength selection threshold of 0.6 are presented in Table 7. For prolamin content prediction, the BPNN model demonstrated the best predictive performance, achieving correlation coefficients above 0.798 across all datasets (the training, validation, and test sets). PLSR showed comparatively weaker results: it still maintained correlation coefficients exceeding 0.7 for all datasets. The SVR and CNN models demonstrated inferior performance. For glutelin content prediction, the BPNN model consistently achieved the best predictive performance, followed by the CNN, SVR, and PLSR models in descending order of effectiveness.

Table 8 presents the regression analysis results of the PLSR, SVR, BPNN and CNN models using Grad-CAM++-selected characteristic wavelengths with an importance threshold of 0.7. For prolamin content, the BPNN model demonstrated the optimal prediction performance, achieving correlation coefficients above 0.78 across all datasets (the training, validation, and test sets). In comparison, the CNN, SVR, and PLSR models showed relatively inferior predictive capability. For glutelin content, the BPNN model also achieved relatively better results, with correlation coefficients exceeding 0.8 across all the three datasets (the training, validation, and test sets). The CNN, SVR, and PLSR models exhibited relatively poor performance.

Evidently, as the threshold value increased, the number of selected characteristic wavelengths decreased. The results in Table 7 and Table 8 revealed that, for both prolamin and glutelin content prediction, the models in Table 7 generally outperformed their counterparts in Table 8. Although setting a relatively higher threshold included fewer wavelength bands, it may inadvertently exclude other bands containing important information. The results from Table 7 and Table 8 demonstrated that Grad-CAM++ could effectively identify the characteristic wavelengths in CNN models for predicting the prolamin and glutelin content. However, further research should be conducted to determine the optimal importance evaluation metrics and optimal threshold value for wavelength feature selection.

### 3.4. Comparison Between Full-Spectra Models and Feature-Wavelength-Based Models

Overall, the results from both the full-spectra models and feature-wavelength-based models demonstrated that either approach (full spectra and feature wavelengths) could be effectively applied for predicting the rice prolamin and glutelin content. The results presented in the aforementioned tables demonstrated that, for predicting both the prolamin and glutelin content, certain feature-wavelength-based models achieved a comparable or even superior performance to full-spectra models. The full-spectra BPNN model for prolamin prediction showed equivalent results to the SPA-based BPNN model. These results, which varied depending on the models and feature wavelength selection methods, illustrated the importance of choosing an appropriate feature wavelength selection algorithm as well as a model.

Furthermore, the characteristic wavelength selection reduced the number of inputs to the model, with the characteristic wavelength selection reducing the variable inputs by a minimum of 51% (GradCAM++ for prolamin) and a maximum of 88% (CARS for glutelin) in the present study compared to the full spectra. Therefore, overall, the characteristic wavelength selection had some advantages and further validated the feasibility of the characteristic wavelength selection.

## 4. Discussion

This study illustrated the feasibility of using hyperspectral as well as feature selection algorithms combined with machine-learning and deep-learning techniques for the detection of rice prolamin and glutelin content. Prolamin and glutelin are the main proteins of rice. In a previous study, Chen et al. [12] used near-infrared spectroscopic detection to investigate the prediction of protein content in rice flour, including prolamin and glutelin. The results of the study found that the correlation coefficients for the prediction of the content of prolamin and glutelin were 0.94 and 0.97, respectively, which were slightly higher than the prediction results of the present study. However, Chen et al.’s study [12] was conducted on milled rice flour, while we sampled the whole rice with husk, which can be affected by the rice seed samples themselves as well as the husk. This issue needed to be further investigated.

In this study, four modeling approaches, including PLSR, SVR, BPNNs, and CNNs, were used for modeling studies. Overall, the BPNN model demonstrated superior predictive performance for both prolamin and glutelin content, regardless of whether full-spectra or characteristic-wavelength-based modeling approaches were employed. This phenomenon is consistent with our previous study on hyperspectral imaging to detect the milling quality in rice [28]. The spectral data used in this study were the same as those in our previous paper, and the comparison results of the CNN and BPNN performance were also consistent. The main reason might be that the dataset in this study was relatively small to allow the CNN to effectively extract the features for predicting the protein content. CNNs typically require a larger sample size to achieve a better predictive performance. Another possible reason was that the pattern of the spectral data in this study was better-suited for fitting by the BPNN. Convolution operations in CNNs process the current feature and adjacent features, focusing on local feature extraction, while BPNNs process the combination of all input features. These factors might explain why the prediction results of CNNs were not as good as those of BPNNs. Whereas the results of the CNN model in this study are not the best, this phenomenon also exists in other studies [28,38,39,40]. These phenomena may be related to the size of the sample set, the structure and distribution of the spectral data, and the way different models extract features and learn, as well as the different parameter settings and optimization approaches [28,38]. PLSR and SVR were commonly used conventional machine-learning methods, and interactive validation was a commonly used method for parameter optimization. They could handle the small dataset well, by extracting the important features for the tasks, which were also easily implemented. However, these two methods might not be able to dig into the deep features in the spectral data, resulting in a relatively limited modeling performance in this study. BPNNs and CNNs were generally modeled based on the training set, and then evaluated for their predictive performance based on the results of the validation sets. Both BPNNs and CNNs were neural network-based models, and they were able to exact the shallow and deep features. However, the model structure might be more complex, which might hinder their applications on the spectra data with simple patterns. Thus, in this study, the performance of models varied on different datasets. In addition, a phenomenon that should be noticed was that there was overfitting in some models, while the results of the other models did not show overfitting. The reason might be attributed to the data distribution and the model principles. Different models processed the sample data in different patterns, and the data distributions could affect the model performance. This phenomenon indicated that further exploration with more samples and more diverse protein content should be conducted.

In this study, SPA, CARS, and GradCAM++ were used for feature wavelength selection. The feature wavelengths selected by SPA and CARS were roughly consistent. It could be found that the feature wavelengths selected by GradCAM++ had some of the same wavelengths as SPA and CARS. The reason might be that a continuous range of spectral wavelengths set by a threshold was selected by GradCAM++ in this study, which covered some of the wavelengths selected by SPA and CARS. As for Gradcam++, the results of the prediction models based on the feature wavelengths selected by different thresholds were acceptable and close to the models built based on the feature wavelengths selected by other methods. These results indicated that it was feasible for GradCAM++ to be used for feature wavelength selection. However, how to set the threshold for feature wavelength selection based on GradCAM++ was also important and needed further research.

SPA conducts variable projection to form different candidate subsets with different wavelengths, and the performance of these subsets was evaluated by constructing the models using these subsets. CARS is a method based on PLSR, and it eliminates variables during the selection procedure, and PLSR models are built using the remaining variables for comparison. This procedure lasts until the expected results are achieved. Based on the optimal CNN models, GradCAM++ calculates the weight of each wavelength for the task of the sample, and it is a post-selection method. These differences in algorithmic principles contributed to the differences in the selected feature wavelengths. Both SPA and CARS selected discrete features, while GradCAM++ selected continuous wavelengths in certain spectral regions. SPA and CARS were widely used feature wavelength methods, and GradCAM++ was rarely used. As mentioned above, models using wavelengths selected by SPA, CARS, and GradCAM++ could obtain acceptable results, indicating the effectiveness of the three feature wavelength selection methods. Since GradCAM++ was based on CNNs, although, in this study, CNN models did not obtain the best performance, the recent studies have shown that the deep-learning models outperformed the conventional machine-learning models in many studies [41]. Thus, the deep-learning-based GradCAM++ has great potential in feature wavelength selection.

As for the identified important wavelengths, the wavelengths in the range of 950–1000 nm [42], and 1300–1600 nm [43] might be attributed to the absorption of OH groups. The wavelengths in the range of 1000–1100 nm might relate to the second overtones for NH bonds, and the wavelengths in the range of 1450–1550 nm might also be attributed to the first overtones of NH bonds [44]. The wavelengths in the range of 1100–1250 nm might relate to the second overtones for CH bonds, while the wavelengths between 1600 nm and 1684 nm might be assigned to the first overtones of CH bonds [44].

These data analysis strategies were developed to predict the content of prolamin and glutelin in rice as accurately as possible, which is very useful for breeding and quality inspection. In actual rice consumption, the difference between the 0.02 and 0.03 protein unit of different rice samples does not actually have a significant impact on its nutritional value and commercial value. However, if the difference is larger, especially when it is more significant, it will have an impact and cause significant quality differences, thereby affecting its commercial value. Previous studies have demonstrated that a higher prolamin content or ratio of prolamin to glutelin (prolamin/glutelin) deteriorates the rice-eating quality which directly affects the rice market and consumer’s choice [3,45]. On the other hand, the overall results indicated that the prediction performance should be improved in the future by using more samples and covering more sample diversity. However, in this study, although the different nutritional treatments and different rice varieties were adopted in order to introduce more sample variations, more biological, physical, and chemical variations should be included in the samples to improve the model performance and model generalization ability.

As mentioned above, it should be noted that there is still a long way to bring the research for real-world application. However, the results of this research also demonstrated the great potential for the rapid, non-destructive, and accurate detection of prolamin and glutelin content in rice in real-world scenarios. In actual production, it is only necessary to spread the seeds without stacking them, eliminating the need for individual separation, to obtain the average spectrum of all seeds. The entire data collection and processing procedure is highly efficient. When the model incorporates more biological and environmental variations and demonstrates robust and excellent results, further research can be conducted in order to advance its industrial application for assessing the rice quality, which holds significance for breeding and enhancing the commercial value of rice, among other related aspects. These directions represent the focus of future research efforts. This study investigated the prediction of the prolamin and glutelin content in rice using hyperspectral imaging technology and data analysis methods. Although the overall results were acceptable, the current model accuracy is still insufficient, and the limited number of samples reduces its generalizability, leaving a gap between the research and practical application. In future studies, it will be necessary to examine more samples and explore different rice forms (intact rice seed, different degrees of milling, and flour with varying particle sizes), as well as to develop data analysis methods and strategies for building more accurate, stable, and generalizable models. The data augmentation strategies should also be explored in order to increase the number of samples to improve the model accuracy, robustness, and generalization ability.

## 5. Conclusions

This study successfully predicted the prolamin and glutelin content in rice by combining hyperspectral imaging technology with machine-learning and deep-learning algorithms, further integrated with feature wavelength selection. For the prediction of the prolamin and glutelin content, among the detection models based on the full spectra and feature wavelengths, the BPNN model achieved the best predictive performance, while the CNN, SVR, and PLSR models exhibited varying results under different conditions. The full-spectra-based model and the feature-wavelength-based model exhibit different prediction results. The feature-wavelength-based models achieved comparable or even better results than the full-spectra-based models, demonstrating the great potential of feature wavelength selection in predicting the prolamin and glutelin content, as well as the feasibility of using GradCAM++ for feature wavelength selection. Meanwhile, these results varied depending on the model and the feature wavelength selection method. Overall, the findings indicated that selecting appropriate modeling methods and feature wavelength selection strategies was crucial for the prediction of the prolamin and glutelin content. This study demonstrated that hyperspectral imaging technology combined with data analysis could be applied in order to predict the prolamin and glutelin content in rice. However, the overall prediction accuracy of this research was not sufficiently high to meet the requirements of practical applications. The limitations of the study included an insufficient sample size, which prevented deep-learning algorithms from learning adequate features. The model should also incorporate more biological and environmental variations to improve its robustness. If these limitations are overcome with promising results and high robustness, further research can be conducted to advance its industrial application for rice quality assessment, which holds significance for breeding and enhancing the commercial value of rice. These directions represent the key focuses for future research efforts, helping to promote the potential applications in the food industry. In future research, we will collect more diverse and representative samples, investigate data analysis methods and factors influencing the model accuracy, further improve the prediction accuracy of prolamin and glutelin content in rice, and promote the practical application of related technologies and models.

## Figures and Tables

**Table 1:** Statistical information of prolamin and glutelin content in the three datasets (units: %).

		Minimum	Maximum	Mean	Standard Deviation
Prolamin	Training	0.51	0.88	0.70	0.07
	Validation	0.55	0.83	0.70	0.07
	Test	0.51	0.85	0.69	0.08
Glutelin	Training	3.43	7.38	5.01	0.99
	Validation	3.32	6.74	4.91	1.02
	Test	3.27	7.00	5.07	1.03

**Table 2:** Prediction results of prolamin and glutelin content using PLSR, SVR, BPNN, and CNN models based on full spectra (units of RMSEC, RMSEV and RMSEP: %).

Model	Label	Training		Validation		Test	
		*r*_c_ ^a^	RMSEC	*r*_v_	RMSEV	*r*_p_	RMSEP
PLSR	Prolamin	0.748	0.048	0.753	0.046	0.722	0.057
	Glutelin	**0.858**	**0.503**	0.829	0.578	0.794	0.667
SVR	Prolamin	0.641	0.056	0.649	0.051	0.714	0.059
	Glutelin	0.856	0.509	0.811	0.599	0.828	0.598
CNN	Prolamin	0.726	0.093	0.740	0.968	0.779	0.103
	Glutelin	0.842	1.461	0.876	1.462	0.813	1.410
BPNN	Prolamin	**0.855**	**0.038**	**0.809**	**0.043**	**0.831**	**0.051**
	Glutelin	0.849	0.517	**0.880**	**0.492**	**0.902**	**0.526**

^a^: *r*_c_, *r*_v_, and *r*_p_ are the correlation coefficients for the training set, validation set, and test set, respectively. RMSEC, RMSEV, and RMSEP are the RMSE values for the training set, validation set, and test set, respectively. The results in bold indicate that optimal results of each type of protein in the training, validation, and test sets among the four models. The results marked in yellow indicated the overall optimal models for each type of protein.

**Table 3:** Results of characteristic wavelength selection for prolamin and glutelin using the SPA algorithm.

Attributes	Number	Wavelengths (nm)
Prolamin	30	981, 1005, 1019, 1033, 1057, 1071, 1085, 1092, 1113, 1127, 1144, 1165, 1193, 1249, 1326, 1358, 1383, 1393, 1397, 1404, 1407, 1414, 1428, 1439, 1446, 1474, 1499, 1577, 1648, 1680
Glutelin	30	981, 1001, 1019, 1033, 1057, 1074, 1085, 1102, 1127, 1137, 1147, 1196, 1266, 1326, 1358, 1383, 1400, 1404, 1407, 1411, 1414, 1425, 1436, 1439, 1446, 1474, 1499, 1535, 1613, 1681

**Table 4:** Regression analysis results of PLSR, SVR, BPNN, and CNN models based on SPA-selected characteristic wavelengths.

Model	Label	Training		Validation		Test	
		*r*_c_ ^a^	RMSEC	*r*_v_	RMSEV	*r*_p_	RMSEP
PLSR	Prolamin	0.708	0.051	0.714	0.049	0.706	0.059
	Glutelin	0.827	0.551	0.803	0.602	0.726	0.726
SVR	Prolamin	0.638	0.056	0.644	0.052	0.730	0.057
	Glutelin	0.829	0.548	0.808	0.595	0.748	0.705
CNN	Prolamin	0.775	0.059	0.791	0.052	0.812	0.057
	Glutelin	0.817	0.656	0.836	0.646	0.835	0.732
BPNN	Prolamin	**0.898**	**0.032**	**0.810**	**0.040**	**0.830**	**0.048**
	Glutelin	**0.995**	**0.089**	**0.875**	**0.497**	**0.920**	**0.409**

^a^: *r*_c_, *r*_v_, and *r*_p_ are the correlation coefficients for the training set, validation set, and test set, respectively. RMSEC, RMSEV, and RMSEP are the RMSE values for the training set, validation set, and test set, respectively. The results in bold indicate that optimal results of each type of protein in the training, validation, and test sets among the four models. The results marked in yellow indicated the overall optimal models for each type of protein.

**Table 5:** Results of characteristic wavelength selection for prolamin and glutelin content prediction using the CARS algorithm.

Attributes	Number	Wavelengths (nm)
Prolamin	24	984, 1022, 1026, 1071, 1088, 1186, 1217, 1252, 1256, 1273, 1291, 1295, 1379, 1389, 1467, 1471, 1503, 1549, 1577, 1599, 1606, 1638, 1642, 1659
Glutelin	44	984, 991, 1040, 1043, 1064, 1074, 1106, 1134, 1151, 1189, 1193, 1207, 1210, 1228, 1242, 1252, 1266, 1288, 1305, 1323, 1340, 1347, 1372, 1376, 1379, 1383, 1411, 1414, 1446, 1453, 1474, 1478, 1485, 1492, 1499, 1513, 1534, 1538, 1542, 1574, 1581, 1613, 1617, 1638

**Table 6:** Regression analysis results of PLSR, SVR, BPNN, and CNN models using CARS-selected characteristic wavelengths.

Model	Label	Training		Validation		Test	
		*r*_c_ ^a^	RMSEC	*r*_v_	RMSEV	*r*_p_	RMSEP
PLSR	Prolamin	**0.829**	**0.040**	0.714	0.053	0.677	0.062
	Glutelin	**0.887**	**0.452**	0.791	0.627	0.829	0.628
SVR	Prolamin	0.645	0.056	0.649	0.051	0.695	0.060
	Glutelin	0.862	0.498	0.818	0.587	**0.877**	**0.532**
CNN	Prolamin	0.718	0.053	0.770	**0.045**	0.797	**0.053**
	Glutelin	0.775	0.840	0.867	0.694	0.816	0.901
BPNN	Prolamin	0.754	0.051	**0.798**	0.051	**0.807**	0.062
	Glutelin	0.849	0.519	**0.873**	**0.499**	0.864	0.540

^a^: *r*_c_, *r*_v_, and *r*_p_ are the correlation coefficients for the training set, validation set, and test set, respectively. RMSEC, RMSEV, and RMSEP are the RMSE values for the training set, validation set, and test set, respectively. The results in bold indicate that optimal results of each type of protein in the training, validation, and test sets among the four models. The results marked in yellow indicated the overall optimal models for each type of protein.

**Table 7:** Regression analysis results of PLSR, SVR, BPNN, and CNN models using Grad-CAM++-selected characteristic wavelengths (threshold = 0.6).

Model	Label	Training		Validation		Test	
		*r*_c_ ^a^	RMSEC	*r*_v_	RMSEV	*r*_p_	RMSEP
PLSR	Prolamin	0.716	0.050	0.719	0.048	0.744	**0.055**
	Glutelin	0.845	0.524	0.778	0.648	0.808	0.628
SVR	Prolamin	0.639	0.056	0.660	0.050	0.724	0.060
	Glutelin	0.823	0.560	0.820	0.592	0.798	0.641
CNN	Prolamin	0.678	0.056	0.713	0.054	0.780	0.059
	Glutelin	0.817	0.645	0.844	0.614	0.806	0.650
BPNN	Prolamin	**0.887**	**0.031**	**0.798**	**0.047**	**0.824**	0.056
	Glutelin	**0.984**	**0.174**	**0.884**	**0.472**	**0.898**	**0.471**

^a^: *r*_c_, *r*_v_, and *r*_p_ are the correlation coefficients for the training set, validation set, and test set, respectively. RMSEC, RMSEV, and RMSEP are the RMSE values for the training set, validation set, and test set, respectively. The results in bold indicate that optimal results of each type of protein in the training, validation, and test sets among the four models. The results marked in yellow indicated the overall optimal models for each type of protein.

**Table 8:** Regression analysis results of PLSR, SVR, BPNN, and CNN models using Grad-CAM++-selected characteristic wavelengths (threshold = 0.7).

Model	Label	Training		Validation		Test	
		*r*_c_ ^a^	RMSEC	*r*_v_	RMSEV	*r*_p_	RMSEP
PLSR	Prolamin	0.645	0.055	0.678	0.050	0.753	**0.055**
	Glutelin	0.757	0.641	0.769	0.646	0.687	0.764
SVR	Prolamin	0.636	0.056	0.653	0.051	0.771	**0.055**
	Glutelin	0.692	0.719	0.727	0.731	0.712	0.724
CNN	Prolamin	0.647	0.057	0.709	0.049	**0.791**	0.056
	Glutelin	0.730	0.812	0.772	0.758	0.691	0.917
BPNN	Prolamin	**0.881**	**0.034**	**0.803**	**0.045**	0.787	0.057
	Glutelin	**0.923**	**0.378**	**0.901**	**0.443**	**0.815**	**0.598**

^a^: *r*_c_, *r*_v_, and *r*_p_ are the correlation coefficients for the training set, validation set, and test set, respectively. RMSEC, RMSEV, and RMSEP are the RMSE values for the training set, validation set, and test set, respectively. The results in bold indicate that optimal results of each type of protein in the training, validation, and test sets among the four models. The results marked in yellow indicated the overall optimal models for each type of protein.

## Data Availability

The original contributions presented in the study are included in the article; further inquiries can be directed to the corresponding authors.
